# Functional exchangeability of the nuclear localization signal (NLS) of capsid protein between PCV1 and PCV2 in vitro: Implications for the role of NLS in viral replication

**DOI:** 10.1186/1743-422X-8-341

**Published:** 2011-07-06

**Authors:** Jiangbing Shuai, Linglin Fu, Xiaofeng Zhang, Binglin Zhu, XiaoLiang Li, Yongqiang He, Weihuan Fang

**Affiliations:** 1 Zhejiang Entry-Exit Inspection and Quarantine Bureau, 126 Fuchun Road, Hangzhou 310016, China; 2 Institute of Preventive Veterinary Medicine, Zhejiang Provincial Key Laboratory of Preventive Veterinary Medicine, Zhejiang University, 388 Yu Hang Tang Road, Hangzhou, 310058, China; 3 Food Safety Key Laboratory of Zhejiang Province, School of Food Science and Biotechnology, Zhejiang Gongshang University, 149 Jiao Gong Road, Hangzhou, 310035, China

## Abstract

**Background:**

Porcine circovirus type 2 (PCV2) is believed to be the primary causative agent of postweaning multisystemic wasting syndrome (PMWS). It is supposed that capsid protein of PCV may contribute to replication control via interaction between Cap and Rep in the nucleoplasm. In this study, we described the construction and in vitro characterization of NLS-exchanged PCV DNA clones based on a PMWS-associated PCV2b isolate from China to determine the role of ORF2 NLS in PCV replication.

**Results:**

The PCV1, PCV2, PCV2-NLS1 and PCV1-NLS2 DNA clone were generated by ligating a copy of respective genome in tandem with a partial duplication. The PCV2-NLS1 and PCV1-NLS2 DNA clone contained a chimeric genome in which the ORF2 NLS was exchanged. The four DNA clones were all confirmed to be infectious in vitro when transfected into PK-15 cells, as PCV capsid protein were expressed in approximately 10-20% of the transfected cells. The in vitro growth characteristics of the DNA clones were then determined and compared. All the recovered progeny viruses gave rise to increasing infectious titers during passages and were genetically stable by genomic sequencing. The chimeric PCV1-NLS2 and PCV2-NLS1 viruses had the final titers of about 10^4.2 ^and 10^3.8 ^TCID_50_/ml, which were significantly lower than that of PCV1 and PCV2 (10^5.6 ^and 10^5.0 ^TCID_50_/ml, respectively). When the ORF2 NLS exchanged, the mutant PCV2 (PCV2-NLS1) still replicated less efficiently and showed lower infectious titer than did PCV1 mutant (PCV1-NLS2), which was consistent with the distinction between wild type PCV1 and PCV2.

**Conclusions:**

Recovery of the chimeiric PCV1-NLS2 and PCV2-NLS1 progeny viruses indicate that the nuclear localization signal sequence of capsid protein are functionally exchangeable between PCV1 and PCV2 with respect to the role of nuclear importing and propagation. The findings also reveal that ORF2 NLS play an accessory role in the replication of PCV. However, we found that ORF2 NLS was not responsible for the distinction of in vitro growth characteristic between PCV1 and PCV2. Further studies are required to determine the in vivo viral replication and pathogenicity of the NLS chimeric DNA clones.

## Background

Porcine circovirus (PCV) is the smallest single-stranded DNA virus replicating autonomously relying upon host cell encoded proteins due to limited coding capacity [1]. Two types of PCV have been characterised. PCV1 was identified as the persistent, non-cytopathic contamination of the porcine kidney cell line (PK-15) [2]. However, PCV2 is believed to be the primary causative agent of postweaning multisystemic wasting syndrome (PMWS) [3,4]. The PMWS mainly affects weanling piglets at 3 to 15 weeks age, which has high morbidity and mortality rate of up to 60% and 15% to 20%, respectively [5]. The PMWS is characterized by generalized lymphadenopathy, lymphocytic depletion and multinucleated giant cell formation in lymph nodes, and lymphoid organ destruction, with inflammatory lesions in multiple organs [6]. Three major open reading frames (ORF) are oriented in opposite directions in the genome of PCV. ORF1 encodes the replicase essential for viral DNA replication and is more conserved between the two genotypes [7], whereas the ORF2 is highly variable between PCV1 and PCV2 (less than 60% homology) and encodes the only structural capsid (Cap) protein that contains dominant epitopes [8,9]. Recently, an apoptosis inducing protein encoded by ORF3 in PCV2 was identified, which was not essential for viral replication but expedited the spread of virus [10,11]. Previous studies revealed that both the nonpathogenic PCV1 and the pathogenic PCV2 replicated via rolling cycle replication mechanism proposed for the *Mastrevirus *genus of the *Geminiviridae *family and utilized comparable genetic elements similarly located along their respective genomes [11-15]. Furthermore, presence of a conserved stem-loop structure and identical essential core element at the origin of DNA replication and similarities among ORF1 in genomes of both PCVs indicated that the loop sequences of these two viruses are functionally interchangeable with respect to protein synthesis and DNA replication [16]. Taken together, the findings showed that replication factors of PCV1 and PCV2 were functionally exchangeable and the replication strategy may not be the main factor determining the distinct propagation efficiency and pathogenicity of PCV1 and PCV2 [17].

Since DNA synthesis of circoviruses occurs exclusively in the nucleus, such macromolecules can only enter the nucleus during transient nuclear envelope breakdown or when mediated by nuclear localization signals [18-20]. In species of *geminiviridae *which are closely related to PCV, the inactivation of nuclear localization of coat protein result in a drastic reduction in viral ssDNA accumulation, indicating that the coat protein may play a role in controlling the copy number of viral DNA [21-23]. In the case of circoviruses, like PCV and beak and feather disease virus (BFDV), capsid protein is expressed late in the infection cycle and co-located in nucleoplasm together with replication protein, indicating that, beyond encapsidation, capsid protein may contribute to replication control via interaction between Cap and Rep in the nucleoplasm [23,24]. Considering the responsibility of NLS for nuclear accumulation of PCV capsid protein, the information showed above suggested a possible accessory role of the NLS in viral replication, which probably resulted in the difference of growth characteristics between PCV1 and PCV2. Previous studies have demonstrated that the N-terminus of capsid protein PCV1 and PCV2 contains distinct nuclear localization signals which are responsible for the nuclear targeting of capsid protein during the viral life cycle [25,26]. This study describes the construction of infectious clones by mutagenesis on PCV ORF2 NLS and their in vitro characteristics to determine the exchangeability of PCV ORF2 NLS and its influence on viral replication and propagation.

## Methods

### Cells and virus stocks

The PK-15 cell lines free or contaminated of PCV1 are propagated in MEM media supplemented with 8% fetal bovine serum (FBS, Gibco) at 37°C with 5% CO_2_. The PCV1 and PCV2b virus stock were originated from the contaminated PK-15 cell line and isolated from tissue sample of a pig with naturally occurring PMWS [27].

### Construction of PCV1 and PCV2 DNA clones

**Figure 1:**
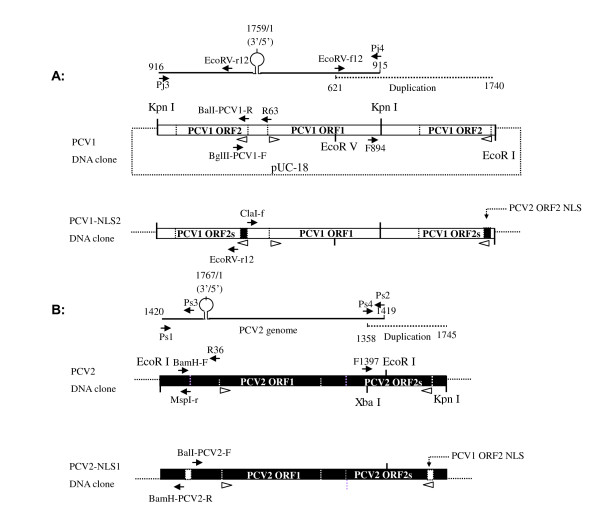
**Schematic construction of the infectious DNA clones**. (A) The PCV1 DNA clone was constructed by ligating a duplicated fragment (915-1740 nt) to the *Kpn *I-digested PCV1 genome in pUC-18. The chimeric PCV1-NLS2 DNA clone was constructed replacing the ORF2 NLS sequence in PCV1 genomic backbone with NLS of PCV2 ORF2. (B) The PCV2 DNA clone was constructed by ligating a fragment (1419-1745 bp) to the *Eco*R I-digested PCV2 genome in pUC-18. The chimeric PCV2-NLS1 was constructed by replacing the PCV2 ORF2 NLS with PCV1 ORF2 NLS. The partial duplications were indicated by dashed. Positions of primers and the directions of ORF were marked by arrows and the orientations of the triangles, respectively.

Non-pathogenic PCV1 and pathogenic PCV2b viral DNA were extracted respectively from PCV1-persistently contaminated or PCV2-propagated PK-15 cells using the QIAamp DNA Mini kit (QIAGEN) according to the manufacturer's instruction. PCV1 complete genome with an overlapping region containing the unique *Kpn I *restriction site was amplified with primers Pj3 and Pj4 (Table 1) designed based on the published sequence of PCV1 [Genbank: NC006266]. The expected product with 1759 bp was separated by gel electrophoresis, purified, digested by *Kpn I *restriction enzyme, and then cloned into pUC-18 vector, resulting in plasmid pUC-PCV1. Another pair of primers EcoRV-f12/EcoRI-r12 amplified a DNA fragment of PCV1 genome (nt 621-1740) encompassed by *EcoR V *and *EcoR I *restriction enzymes. Both the purified fragment and pUC-PCV1 were excised with *EcoR V *and *EcoR I *and then joined at the restricted sites. The resultant PCV1 DNA clone pIS-PCV1 carried a complete genome and a duplicated fragment of PCV1 from 916 nt to 1740 nt (Figure 1).

**Table 1:** Primers used in construction of DNA clones.

DNA clones	Primer	Primer Sequences (5'-3')^c^	Primer location	Application
PCV2	Ps1	GCGAATTCAACCTTAACCTTTCTTATTCT	1418-1446 nt	PCV2 genome
DNA clone	Ps2	AAGAATTCTGGCCCTGCTCCC	1407-1427 nt	
	^a ^Ps3	GCTCTAGAATAACAGCACTGGAG	1356-1378 nt	Duplicate
	^a ^Ps4	GAGGTACCCGTTTTCAGATATGAC	1730-1753 nt	
				
PCV1	Pj3	ACGGTACCCGAAGGCCGATTTGAA	914-937 nt	PCV1 genome
DNA clone	Pj4	TAGGTACCTCCGTGGATTGTTCTCC	899-923 nt	
	^b^EcoRV-f12	GCGATATCATGGAGAAGAAGTTGTTGT	649-675 nt	Duplicate
	^b^EcoRI-r12	CCGAATTCTCTTTCACTTTTATAGGATG	1721-1748 nt	
				
PCV1-NLS2	ClaI-f	CAATCGATAACGCCTCCTTGGATACGTCATC	1694-1724 nt	PCV1 ΔNLS
DNA clone	BglII-r	GGAGATCTTCAATTCCCGCCTTTCTAG	1566-1592 nt	
	BamH-F	TAGGATCCCCGTGCCATTTTTCCTTC	1594-1619 nt	PCV2 NLS
	MspI-r	CGTTACCGGAGAAGAAGACAC	1696-1716 nt	
				
PCV2-NLS1	BalI-PCV2-F	CCTTGGCCACGTCATATCTGAA	1720-1741 nt	PCV2 ΔNLS
DNA clone	BamH-PCV2-R	GAAAAATGGGATCCTCAACACC	1594-1615 nt	
	BglII-PCV1-F	TACAGATCTTTCGGCGCCA	1588-1606 nt	PCV1 NLS
	BalI-PCV1-R	TATTGGCCAAGGAGGCGTTA	1701-1720 nt	

a, Primers also used for amplification of duplicate in PCV2-NLS1 DNA clone construction.

b, Primers also used for amplification of duplicate in PCV1-NLS2 DNA clones construction.

c, The restriction enzyme sites were underlined.

For PCV2 DNA clone construction, a pair of primers (Ps1 and Ps2) was designed according to PCV2 SH strain [Genbank:DQ195679] for amplification of PCV2 complete genome encompassed with the unique *EcoR I *restriction site which was then digested and ligated within pUC-18 as a recombinant plasmid pUC-PCV2. An overlapped fragment (nt 1358-1745) of PCV2 genome was amplified with Ps3/Ps4 containing *Xba I *and *Kpn I *restriction region. After digestion with the enzymes, the fragment and linearized pUC-PCV2 DNA were ligated, resulting in the PCV2 DNA clone pIS-PCV2 which included a full-length PCV2 genome and a duplicated fragment from 1419 nt to 1745 nt.

### Construction of chimeric PCV2-NLS1 and reciprocal PCV1-NLS2 DNA clones

To construct PCV2-NLS1 DNA clone containing chimeric PCV2-NLS1 genome in which NLS sequence of PCV2 ORF2 was replaced by that of PCV1, the whole recombinant vector sequence without PCV2 ORF2 NLS was amplified from pUC-PCV2 with primer pair BalI-PCV2-F/BamH-PCV2-R and introduced restriction enzyme sites of *Bal I *by point mutation and *BamH I*. After digestion, the linearized vector sequence was ligated with PCV1 NLS sequence which was amplified with primers of BglII-PCV1-F and BalI-PCV1-R and digested with *Bgl II *and *Bal I*, resulting in vector pUC-PCV2-NLS1 containing chimeric PCV2-NLS1 genome. The genome was then cut from pUC-PCV2-NLS1 by *EcoR I *and re-cyclized. The duplicated fragment (nt 1358-1745) was amplified with Ps3/Ps4 from the circular genome, digested with *Xba I *and *Kpn I *and subcloned into pUC-PCV2-NLS1, as similar to the construction of PCV2 DNA clone. The consequent DNA clone pIS-PCV2-NLS1 comprised a full-length and a duplicated fragment from 1419 nt to 1745 nt of the chimeric PCV2-NLS1 genome.

To construct a reciprocal PCV1-NLS2 chimeric DNA clone, the nuclear signal sequence of PCV1 ORF2 was replaced by that of PCV2. Briefly, the PCV2 ORF2 NLS sequence was amplified by primers of BamH-F/MspI-r and ligated into recombinant plasmid sequence without PCV1 ORF2 NLS amplified from pUC-PCV1 by ClaI-F and BglII-r. The resultant pUC-PCV1-NLS2 plasmid was then digested with *Kpn I *and the chimeric PCV1-NLS2 genome was re-cyclized. A duplicated fragment was amplified with EcoRV-f12/EcoRI-r12 from the circular genome, digested with *EcoR V *and *EcoR I *and subcloned into pUC-PCV1-NLS2, resulting in the consequent PCV1-NLS2 DNA clone pIS-PCV1-NLS2.

### Transfection of PK-15 cells with PCV1, PCV2 and PCV2-NLS1 DNA clones

To evaluate the in vitro infectivity of cells, the DNA clones were purified by using Qiagen plasmid mini kit (Qiagen, USA) and were transfected into PCV free PK-15 cells with Lipofectamine™ 2000 (Invitrogen, USA) according to the manufacturer's protocols. Briefly, 80% confluent cells in 24-well plate were incubated with the mixtures of 0.8 μg each plasmid DNA and 2.5 μl Lipofectamine™ 2000. Mock-transfected cells with pUC-18 vector were included as a negative control. Two days post-transfection, an immunofluorescence assay (IFA) was performed to confirm the infectivity of DNA clones. Cells were fixed with a pre-cooled 80% acetone at -20 °C for 15 minutes. After washing with phosphate-buffered saline (PBS) containing 0.05% Tween-20 (PBS-T), the cells were incubated at 37°C for 1 hour with 1:500-diluted polyclonal antibodies to PCV1 or PCV2 capsid protein. The plate was washed three times with PBS-T and incubated with the secondary fluorescein isothiocyanate-labeled goat anti-rabbit IgG (KPL, USA) at 37°C for another 45 minutes. After washed and mounted, the cells were examined for expression of viral antigen under a fluorescence microscope (Leica DM6000B).

The genomic viral DNA distinguished from the input plasmid DNA was monitored by primers R63 (5'-CTTTTCTTGCTTGGCATT-3', 46-63 nucleotide, nt) and F894 (5'-CTGCTGGAGAACAATCC-3', 894-910 nt) for PCV1 and PCV1-NLS2 as well as primers F1397 (5'-TGGGTGATCGGGGAGCAGG-3', 1397-1415 nt) and R36 (5'-GCTGCCGAGGTGCTGCCGCT-3', 17-36 nt) for PCV2 and PCV2-NLS1.

### Recovery and in vitro viability evaluation of the progeny viruses

In order to determine the viability and in vitro characteristics of the progeny viruses, PK-15 cells at 80% confluence in 6-well plate were transfected with 20 μg of PCV1, PCV2, PCV1-NLS2 and PCV2-NLS1 DNA clone, respectively. Each well of cells was collected 5 days post-transfection, frozen and thawed for three times. Subsequently, the cell lysates were inoculated on fresh PK-15 cells in T-25 flasks, incubated for five days and then serially passaged for 26 times. At each passage, 2 ml of the suspended cells from each progeny virus was collected for titration according to the Reed-Muench method. Briefly, each progeny virus stock was serially 10-fold diluted, and each dilution was inoculated into 8 wells on fresh PK-15 cells seeded on 48-well plates. Mock-inoculated cells were incubated as negative controls. The virus- or mock-infected cells were fixed at 3 days post-inoculation and the 50% tissue culture infective dose (TCID_50_) per ml of progeny viruses were determined by IFA as described above and calculated by Reed-Muench method.

### In vitro growth characteristics of PCV1, PCV2, PCV1-NLS2 and PCV2-NLS1 viruses

Twelve-well plates were seeded of PK-15 cells free of PCV at 5 × 10^5 ^cells per well. When the cells were 80% confluent, sixteen wells of cells for each progeny virus were inoculated with PCV1, PCV2, PCV1-NLS2 and PCV2-NLS1 viruses at a multiplicity of infection (MOI) of 0.1, respectively. After incubation for 1 hour, the inoculum was discarded, and the cells were maintained in MEM with 2% fetal bovine serum. Two wells of cells inoculated with each virus were harvested at intervals of 12 hours and stored until titration, virus quantification and western blot analysis. Finally, the infectious titers of progeny viruses at different time points were determined by IFA as described above. And the genomic copy numbers of viruses in cells were quantified by a TaqMan-based real time PCR. Shortly, viral DNA was extracted using the QIAamp DNA Mini kit (QIAGEN) from cells harvested according to the manufacturer's instruction. Genomic DNA copy numbers for each virus at different time points were determined using PCV1 or PCV2 ORF2-based primer pairs and probes (Table 2) on LightCycler^® ^480 (Roche, Germany) under conditions: 3 min at 50°C, 5 min at 94°C, followed by 40 cycles of 10 s at 94°C and 45 s at 60°C. Ten-fold serially diluted pUC-PCV1 and pIS-PCV2 plasmids were included to generate a standard curve.

**Table 2:** Primers and probes designed for Real-time PCR

Primer	Sequence (5'-3')	Primer location	Targeting
Re-ORF22-F1	CGCTCTGTGCCCTTTGAATAC	1325-1347 nt	
Re-ORF22-R1	GTGAGGGCTGTGGCCTTTGTTAC	1450-1470 nt	PCV2 ORF2
PCV2-probe1	FAM-ATTCTGGCCCTGCTCCCCGAT-TAMRA	1403-1423 nt	
			
Re-PCV1-F	AGGTGATGGGGTCTCTG	1384-1400 nt	PCV1 ORF2
Re-PCV1-R	TACCCCTACCTTTCCAATACTAC	1433-1455 nt	
PCV1-FAM	FAM-ATTCATATTTAGCCTTTCTAAT-TAMRA	1408-1429 nt	

Western bolt analysis was conducted to confirm the evidence and kinetics of viral capsid protein expression at different time points. The cells were sedimented by centrifugation and lysed, the proteins were separated by SDS-PAGE and transferred onto nitrocellulose membranes (Pall Co. Ltd., USA) in Semi-dry transfer cell (Bio-Rad) at 20 V for 45 minutes. The membranes were then blocked with 5% (w/v) skim milk diluted in PBS-T for 2 hours at 37°C. After washing, the membranes were probed with PCV1 or PCV2 capsid protein specific polyclonal antibodies (1:250 dilution) for 1 hour, followed by incubation with horseradish peroxidase conjugated goat IgG (KPL Inc., USA) for 45 minutes. Finally, signals were developed with 4-chloro-1-naphthol and examined.

## Results

### The DNA clones were infectious in transfected PK-15 cells and generated infectious progeny viruses in vitro

**Figure 2:**
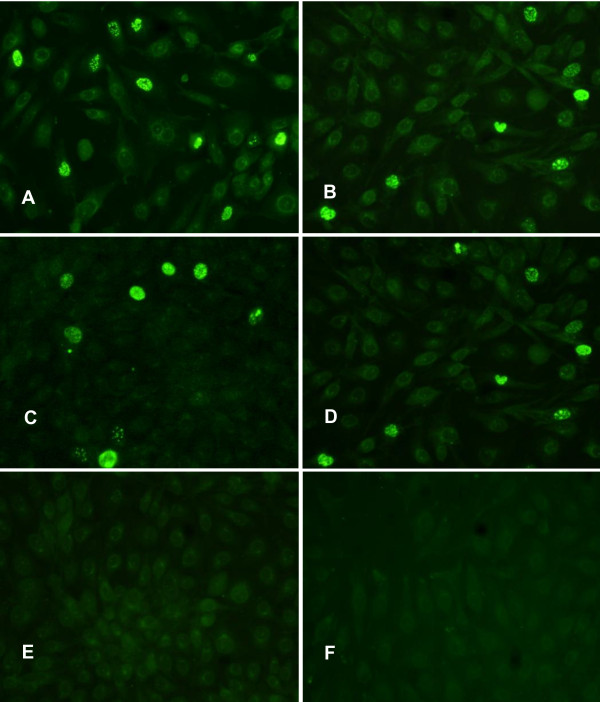
**The DNA clones were infectious when transfected in PK-15 cells in vitro**. The cells were stained with polyclonal antibody to the PCV1 (A and B) or PCV2 (C and D) capsid protein. (A) PK-15 cells transfected with PCV1 DNA clone. (B) PK-15 cells transfected with chimeric PCV1-NLS2 DNA clone. (C) PK-15 cells transfected with PCV2 DNA clone. (D) PK-15 cells transfected with chimeric PCV2-NLS1 DNA clone. (E and F) Mock-transfected PK-15 cells with pUC-18.

PCV1 or PCV2 DNA clone was constructed by ligating a duplicated fragment to the 3' end of respective genome cloned in the pUC-18 vector (Figure 1). Evaluated by IFA two days after transfection, presence of PCV1- or PCV2 capsid protein was observed in approximately 15-20% of the transfected cells (Figure 2A and 2C), whereas the mock-transfected cells with pUC-18 vector remained negative, indicating that the PCV1 and PCV2 DNA clones were infectious in vitro (Figure 2E and 2F).

The PCV1-NLS2 DNA clone contained a chimeric PCV1-NLS2 genome in which NLS sequence of PCV1 ORF2 was replaced by that of PCV2. IFA with PCV1 antibodies confirmed that 10% of the cells were transfected (Figure 2B), showing that the PCV1-NLS2 chimeric DNA clone could replicate in vitro and express chimeric capsid protein with PCV1 ORF2 NLS motifs, as predicted.

In contrast, the reciprocal PCV2-NLS1 DNA clone contained a chimeric PCV2-NLS1 genome in which PCV2 ORF2 NLS sequence was replaced by that of PCV1. The presence of chimeric capsid protein detected in approximately 10% of transfected cells (Figure 2D) indicated that PCV2-NLS1 DNA clone was infectious in vitro and encode chimeric capsid protein with PCV1 ORF2 NLS motifs.

Two primer pairs were introduced to monitor and distinguish the genomic viral DNA produced in transfected cells from the input plasmid DNA. As a result, primers R63 and F894 amplified a fragment of 928 bp which could only be the product from circular genomes of PCV1 or PCV1-NLS2. Moreover, primers F1397 and R36 amplified a 407 bp fragment, which was indicative of the presence of the PCV2 or PCV2-NLS1 viral genome. However, the 4400 bp or 3400 product from the input plasmid DNA was not observed (data not shown). The findings reflected that the capsid proteins detected in transfected cells were synthesized from viral genomes which might be circularized as the result of homologous recombination from DNA clones, rather than from the input plasmid [28].

### Confirmation of the viability and stability of progeny viruses during serial passages

**Figure 3:**
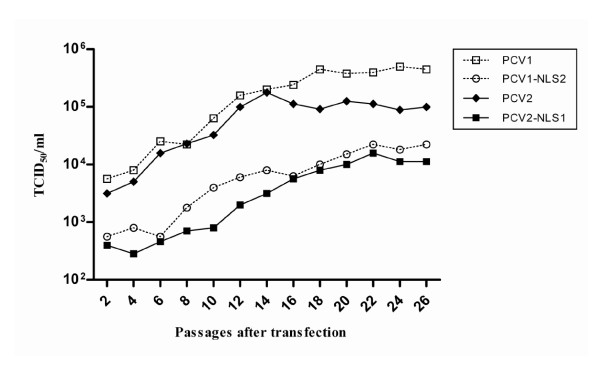
**In vitro viability of progeny viruses after initial transfection of PK-15 cells with DNA clones**. Synchronized PK-15 cells were transfected with 20 μg of PCV1, PCV2, PCV1-NLS2 and PCV2-NLS1 DNA clone followed by twenty-six serial passages. The DNA clones were capable of producing infectious and viable virions in vitro, and all the progeny viruses gave rise to gradually increasing infectious titers during passages and propagated stably after 16 to 20 passages. The infectious titers were determined at each passage according to the Reed-Muench method.

The transfected cell lysates were further inoculated onto fresh PK-15 cells. The viral proteins observed in nucleus of inoculated cells by IFA confirmed that the progeny virions produced by in vitro transfection were infectious. All the homogeneous PCV1, PCV2, PCV1-NLS2 and PCV2-NLS1 virus stocks originated from transfected cells had low initial titers ranged from 10^2.6 ^to 10^3.7 ^TCID_50_/ml, showing no significant difference with each other. However, all the four virus stocks gave rise to gradually increasing infectious titers during passages and propagated stably after 16 to 20 passages (Figure 3), indicating that the DNA clones were capable of producing infectious and viable virions in vitro. The infectious titers of PCV1 and PCV2 maintained at about 10^5.6 ^and 10^5.0 ^TCID_50_/ml respectively, which were significantly higher than that of chimeric PCV1-NLS2 and PCV2-NLS1 (10^4.2 ^and 10^3.8 ^TCID_50_/ml) after adaptation in vitro (*P < 0.05*). However, PCV2-NLS1 had much lower titer than did PCV1-NLS1 (*P < 0.05*), which was consistent with the finding that wild type PCV1 showed statistically higher titer than did PCV2.

The genome sequence of progeny viruses were monitored by genomic sequencing during the passages. The results showed that all the progeny viruses were genetically stable in PK-15 cells for passages, as no additional mutation or reversion was found in the genomes (data not shown). The findings indicated that the nuclear localization signal sequence of ORF2 was functionally exchangeable between PCV1 and PCV2.

### In vitro growth characteristics of PCV1, PCV2, PCV1-NLS2 and PCV2-NLS1 viruses

**Figure 4:**
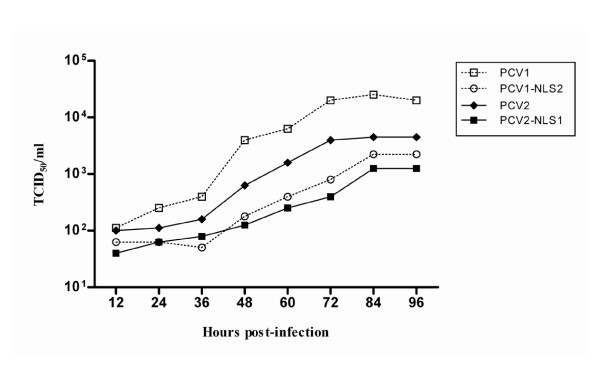
**One-step growth curve of PCV1, PCV2, PCV1-NLS2 and PCV2-NLS1 viruses**. Synchronized PK-15 cells were infected with viruses all at an MOI of 0.1. The infectious titers for all viruses were similarly lower than 10^1.8 ^TCID_50_/ml after initial infection, but gradually increased from 12 to 96 hours. PCV1 propagated much more efficiently in vitro with a significantly higher titer of 10^4.3 ^TCID_50_/ml by 96 h post-infection. The infectious titers of viruses were determined by IFA with Reed-Muench method.

**Figure 5:**
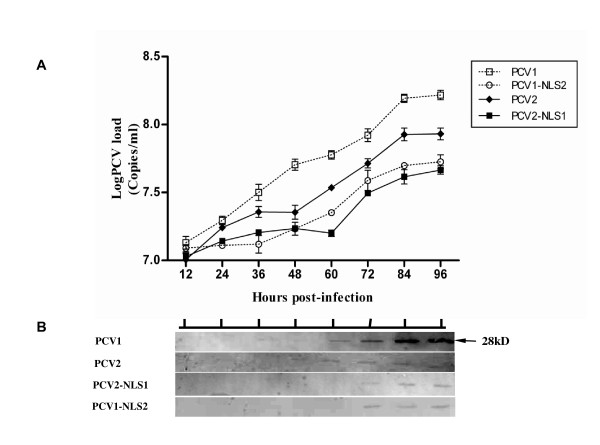
**Synchronized PK-15 cells were infected with viruses all at an MOI of 0.1**. (A)The virus loads for PCV1, PCV2, PCV1-NLS2 and PCV2-NLS1 in cell cultures at each time point were determined by real-time PCR based on PCV1 or PCV2 ORF2 gene. (B) Kinetics of viral capsid protein (28 kD) biosynthesis of progeny viruses from infected PK-15 cells at different time points were confirmed by western blot analysis with polyclonal antibodies to PCV1 or PCV2 capsid protein.

To further confirm the growth characteristics of the progeny viruses in vitro, one-step growth curve was performed for each virus. Cells inoculated with each virus at an MOI of 0.1 were collected at intervals of 12 hours. The titers for each virus at different time points were determined by IFA. PCV1, PCV2, PCV1-NLS2 and PCV2-NLS1 all gave rise to similar numbers of viruses after initial infection, since the initial infectious titers were all less than 10^1.8 ^TCID_50_/ml (Figure 4). However, the infectious titers for all viruses gradually increased from 12 to 96 hours. PCV1 propagated most efficiently in vitro and had a significantly higher titer of 10^4.3 ^TCID_50_/ml by 96 h post-infection, whereas the infectious titer of PCV2 was 10^3.6 ^TCID_50_/ml. With regard to the two chimeric viruses, PCV1-NLS2 gave better growth performance than PCV2-NLS1, as they had the infectious titers of about 10^3.35 ^and 10^3.0 ^TCID_50_/ml by 96 hour post infection, respectively. The results were further confirmed by real time PCR with the findings which showed 0.7 to 1 log of increasing genomic copy number of all the four viruses during the infection (Figure 5A). In spite of the similar copy numbers of genomic DNA after initial infection for all viruses, PCV1 genomic DNA was detected as about 1.6 × 10^8 ^copies/ml by 96 hour post infection, which was statistically higher than that of the other viruses. Moreover, PCV1-NLS2 replicated more efficiently in vitro, as compared with PCV2-NLS1.

Kinetics of capsid protein biosynthesis of the progeny viruses were then determined by western blotting with specific antibodies to PCV1 or PCV2 capsid protein. As showed in Figure 5B, expression of capsid protein from PCV1 infected cells were observed from 36 h post infection, whereas PCV2 capsid protein were detected from 48 h post infection. However, capsid protein of PCV2-NLS1 and PCV1-NLS2 were detected later until 72 h post infection, which would be due to the low level of propagation efficiency of the chimeric viruses.

## Discussion

PMWS has become one of the most economically important and complex disease syndromes in the global swine industry today. Although the etiology and pathogenesis of PMWS appear to be complicated since other viral or non-viral agents are considered as co-factors [29-31], increasing evidences suggest that PCV2 is the principal causative agent [32-35]. Nevertheless, the limit of viral replication efficiency in vitro and the lack of a biologically pure form of virus stock have impeded the study of the role of PCV2 in pathogenesis of PMWS [36]. However, the use of infectious clone contributes to the research on virus replication, pathogenesis and vaccine development by modification at molecular level and offers an opportunity for availability of biologically pure virus stock [28,37]. In this study, we described the constructions of four different infectious clones based on PCV1 derived from contaminated PK-15 cells and PCV2 strain isolated from PMWS-affected piglets from China. The consequent biologically pure progeny virus stocks were then generated by transfection of PK-15 cells with those DNA clones, followed by analysis of in vitro characteristics of the progeny viruses.

It is reported that homologous recombination may take place when identical or similar sequences are present in one molecule [38]. Thus, all the PCV1, PCV2, PCV2-NLS1 and PCV1-NLS2 DNA clone constructed in this work contained a copy of respective genome ligated in tandem with a partial duplication that enabled the genome to circularize out of the recombinant plasmid by homologous recombination and initiate viral replication. The DNA clones were all found to be infectious in vitro when the plasmids were applied onto PK-15 cells, since viral antigen were expressed in 10-20% of the transfected cells. Moreover, a discriminative PCR amplified indicative fragments of the presence of viral genomes, while no input plasmid DNA was detected. Taken together, the findings confirmed that the PCV1, PCV2, PCV2-NLS1 and PCV1-NLS2 viral genome were functionally produced due to homologous recombination from DNA clones, replicated and further synthesized the viral antigen in vitro. After passages, biologically pure and viable progeny viruses were successfully generated for further in vitro or in vivo studies. And the strategy for construction of DNA clone was useful for research on PCV replication, pathogenesis and vaccine development.

It has been shown for geminiviruses that disruption of coat protein synthesis or loss of its nuclear localization resulted in a drastic reduction in single-stranded genomic DNA accumulation [20,21,39]. PCV2 capsid protein is expressed late in the infection cycle and co-located in nucleoplasm together with replication (Rep) protein [22]. Thus, the coat protein may play a role in replication control and might be expected to influence early events including DNA synthesis via interaction with Rep [22,23]. Previous studies have revealed that the nuclear accumulation of PCV1 and PCV2 capsid protein are properly mediated by their distinct NLS motifs [25,26]. Hence, we questioned that whether the NLS of PCV ORF2 would play an accessory role in viral replication due to its contribution to nuclear accumulation of capsid protein. In this study, both the chimeric capsid protein of PCV1-NLS2, PCV2-NLS1 were visualized in the nucleus of the transfected PK-15 cells, similarly to that of the wild type PCV1 and PCV2. Taken together with the recovery of infectious and genetically stable virions, the results indicated that the nuclear localization signal sequence of capsid protein was functionally exchangeable between PCV1 and PCV2 with respect to the role in nuclear importing and viral propagation. Moreover, the infectious titres and the replication efficiency of chimeric PCV1-NLS2 and PCV2-NLS1 were much lower than that of wild type PCV1 and PCV2 after passages for adaptation in vitro, which indicated that ORF2 NLS played an accessory role in the replication of PCV. However, when the ORF2 NLS exchanged, the mutant PCV2 still replicated less efficiently and showed lower infectious titre than did PCV1 mutant, as the same manner between wild type PCV1 and PCV2. The finding demonstrated that ORF2 NLS was not responsible for the distinction of in vitro growth characteristics between PCV1 and PCV2.

Previous studies have described the in vivo pathogenicity of the molecular PCV2 DNA clone of USA strain and European strain [28,40]. Although differences in pathogenicity among PCV2 isolates from PMWS cases have not been clearly elucidated, numbers of substitutions in PCV2 isolates from China have been described [27]. Thus, further study will be carried out to demonstrate the in vivo viral replication, immunogenicity and pathogenicity of the DNA clones of Chinese PCV2b isolate and its NLS chimeric viruses.

## Conclusions

The findings in this work confirmed that the DNA clones were infectious in vitro and were capable of producing infectious, biologically pure and genetically stable virions which was useful for further research on PCV replication, pathogenesis and vaccine development. The recovery of the PCV1-NLS2 and PCV2-NLS1 progeny viruses also indicated that the nuclear localization signal sequence of capsid protein was functionally exchangeable between PCV1 and PCV2 with respect to the role in nuclear importing and viral propagation. The infectious titres and the replication efficiency of chimeric PCV1-NLS2 and PCV2-NLS1 were much lower than that of wild type PCV1 and PCV2 after passages for adaptation in vitro, indicating that ORF2 NLS play an accessory role in the replication of PCV. Interestingly, the findings also demonstrated that ORF2 NLS was not responsible for the distinction of in vitro growth characteristics between PCV1 and PCV2.

## Competing interests

The authors declare that they have no competing interests.

## Authors' contributions

JS designed the whole project, carried out the DNA clone construction, performed data analysis and drafted the manuscript. LF contributed to the construction of DNA clone and in vitro transfection. XZ performed IFA and viral passages. BZ and XLcontributed to the cell culture and performed Western blot analysis. YH preformed real time PCR. WF supervised the project, participated in the design of the study and data interpretation, and helped to draft the manuscript. All authors have read and approved the final manuscript.
